# Novel Biomaterials for Tissue Engineering 2018

**DOI:** 10.3390/ijms19123960

**Published:** 2018-12-09

**Authors:** Emmanuel Stratakis

**Affiliations:** Institute of Electronic Structure and Laser, Foundation for Research and Technology Hellas, Nikolaou Plastira 100, Heraklion, Crete, GR-70013, Greece; stratak@iesl.forth.gr

The concept of regenerating tissues, with properties and functions that mimic natural tissues, has attracted significant attention in recent years. It provides potential solutions for many diseases treatment and other healthcare problems. To fully realize the potential of the approach, it is crucial to have a rational biomaterial design to create novel scaffolds and other material systems suitable for tissue engineering, repair, and regeneration. Research advances in the topic include the design of new biomaterials and their composites, the scaffold fabrication via subtractive and additive manufacturing approaches, the development of implantable scaffolds for disease monitoring, diagnostics, and treatment, as well as the understanding of cell–biomaterial scaffolds interaction.

In the Special Issue “Novel Biomaterials for Tissue Engineering 2018”, promising findings on different approaches to designing and developing new biomaterials, biomaterial systems, and methods for tissue engineering, are presented and discussed.

In particular, Rahali et al. report on the synthesis and characterization of novel gelatin methacrylate hydrogels functionalized with nanoliposomes and nanodroplets [1]. This nanofunctionalization approach enables control over the design of the biomaterial, via tailoring the type of incorporated nanoparticle for the specific application. Furthermore, hydroxyapatite (HA) films with minute amounts (ca. 1 weight %) of (rhenium-doped) fullerene-like MoS_2_ nanoparticles (IF) were deposited through an electrophoretic process [2]. Tribological tests revealed that the nanoparticles endow the HA film very low friction and wear characteristics. As a consequence, HA-IF films could be of interest for various medical technologies. Al-Kattan et al. [3] reviewed the application of bare, ligand-free, laser-synthesized nanoparticles of Au and Si as functional modules (additives) in scaffold platforms intended for tissue engineering purposes. In addition, a new biodegradable medical adhesive material was recently developed by blending Poly(lactic acid) (PLA) with Poly(trimethylene carbonate) (PTMC) in ethyl acetate [4]. It is shown that in addition to having a positive effect on hemostasis and no sensibility to wounds, PLA-PTMC can efficiently prevent infections. Moreover, Babaliari et al., demonstrated the use of ultrafast laser-fabricated microstructured culture substrates on different materials, including Si, Polyethylene terephthalate (PET), and Poly(lactide-*co*-glycolide) (PLGA), as a mean to control cellular adhesion and orientation [5]. This property is potentially useful in the field of neural tissue engineering and for microenvironment systems that simulate in vivo conditions. The recent achievements in the field of non-apatitic calcium phosphate materials (CaPs) substituted with various ions were reviewed by Laskus and Kolmas [6]. The authors focused particularly on tricalcium phosphates (TCP) and “additives” such as magnesium, zinc, strontium, and silicate ions, all of which have been widely investigated thanks to their important biological role. The review also highlights some of the potential biomedical applications of non-apatitic substituted CaPs. Besides this, Khan and Tanaka [7] discussed the prospect of using functional biomaterials, which respond to external stimulus, for the development of smart 3D biomimetic scaffolds. The authors elaborated on how smart biomaterials are designed to interact with biological systems, for a wide range of biomedical applications, including the delivery of bioactive molecules, cell adhesion mediators, and cellular functioning for the engineering of functional tissues to treat diseases.

Human mesenchymal stem cells (MSCs) have been widely studied for therapeutic development in tissue engineering and regenerative medicine. However, directing the differentiation of MSCs still remains challenging for tissue engineering applications. To address this issue, Balikov et al. [8] developed a low-cost, scalable, and effective copolymer film to direct angiogenic differentiation of MSCs. hMSCs were cultured over several passages without the loss of reactive oxygen species handling or differentiation capacity. In another approach, Lee et al. [9] developed in situ cross-linkable gelatin−hydroxyphenyl propionic acid hydrogels that can direct endothelial differentiation of MSCs, thereby promoting vascularization of scaffolds towards tissue engineering and regenerative medicine applications in humans. Besides this, the development of techniques and devices for the development of new cellular microenvironments (i.e, niche), which is poorly represented by the typical plastic substrate used for the two-dimensional growth of MSCs in a tissue culture flask, is of critical importance. Aubert et al. [10] presented a collagen-based medical device as a mimetic niche for MSCs with the ability to preserve human MSC stemness in vitro. Nativel et al. [11] reported on the application of droplet millifluidics to encapsulate and support viable hMSCs in a polysaccharide hydrogel.

This Special Issue also presents recent advances in bone tissue engineering and regeneration as well as in osteogenic differentiation. Specifically, resorbable bacterial cellulose membranes, treated by electron beam irradiation, have been reported to be excellent biomaterials for guided bone regeneration [12]. Moreover, Hum et al. [13] developed highly porous bioactive glass-based scaffolds, fabricated by the foam replica technique and coated with collagen. The combination of bioactivity, mechanical competence, and cellular response makes this novel scaffold system attractive for bone tissue engineering. In another approach, Hsieh et al. [14] explored the development of solid biomimetic scaffolds of Poly(ε-Caprolactone)/Hydroxyapatite and Glycidyl-Methacrylate-Modified Hyaluronic Acid. In vivo experiments on the healing of osteochondral defects, performed on the knees of miniature pigs, concluded that the structural design of the scaffold should be reconsidered to match the regeneration process of both cartilage and subchondral bone. Besides this, different approaches to osteogenic differentiation are reported. In particular, the osteogenic differentiation effect of the FN Type 10-Peptide Amphiphile (FNIII10-PA) on Polycaprolactone fibers has been investigated [15]. It is shown that the FNIII10-PA-induced the osteogenic differentiation of MC3T3-E1 cells, indicating its potential as a new biomaterial for bone tissue engineering applications. Sobreiro-Almeida et al. investigated the hMSCs osteogenic differentiation on piezoelectric Poly(vinylidene fluoride) microsphere substrates [16]. It is concluded that such microspheres are a suitable support for bone tissue engineering purposes, as hMSCs can proliferate, be viable, and undergo osteogenic differentiation when chemically stimulated. Finally, Paun et al. developed 3D magnetic structures, fabricated by direct laser writing via two-photon polymerization and coated with a thin layer of collagen-chitosan-hydroxyapatite-magnetic nanoparticles composite [17]. In vitro experiments showed that such scaffolds stimulate the osteogenesis in the static magnetic field, via promotion of the MG-63 osteoblast-like cells proliferation and differentiation.

Tissue engineering methods to address skin regeneration and fertility restoration have been additionally reported. Specifically, Pang et al. [18] evaluated the effects of total flavonoids from *Blumea balsamifera* (L.) DC. on skin excisional wounds on the backs of Sprague-Dawley rats and revealed its chemical constitution, as well as its action mechanism. The study concluded that flavonoids were the main active constituents that contribute to excisional wound healing. Del Vento et al., reviewed the tissue engineering approaches to the improvement of immature testicular tissue and cell transplantation outcomes [19]. It is concluded that such bioengineering techniques may be a step closer to fertility restoration for prepubertal boys exposed to gonadotoxic treatments.

Finally, this Special Issue includes recent advances in biofabrication techniques for tissue engineering purposes. In particular, Zhang et al., provided an overview of the application of the layer-by-layer (LbL) self-assembly technology for the surface design and control of biomaterial scaffolds to mimic the unique features of native extracellular matrices [20]. It is concluded that LbL self-assembly not only provides advances for molecular deposition but also opens avenues for the design and development of innovative biomaterials for tissue engineering. Another emerging biofabrication tool is 3D printing, which has been recently applied for the development of an artificial trachea [21]. It is shown that epithelial cells in the 3D bioprinted artificial trachea were effective in respiratory epithelium regeneration. Furthermore, chondrogenic-differentiated bone marrow-derived MSCs had more neo-cartilage formation potential in a short period, although in a localized area. Furthermore, Park et al. [22] demonstrated the generation of oriented ligamentous architectures, driven by a 3D-printed microgroove pattern. The results of this study demonstrate that 3D-printed topographical approaches can regulate spatiotemporal cell organizations that offer strong potential for adaptation to complex tissue defects to regenerate ligament-bone complexes. In another study, a new bone substitute developed from 3D-printed structures of Polylactide (PLA) loaded with collagen I, have been demonstrated [23]. The results obtained from in vitro cultures of various cell types, including osteoblasts, osteoblast-like, fibroblasts, and endothelial, indicate the potential use of 3D-printed PLA scaffolds in bone tissue engineering. Being a promising biofabrication technique, electrospinning has been widely used for the fabrication of extracellular matrix (ECM)-mimicking fibrous scaffolds for several decades. In this context, Jun et al. [24] summarized the fundamental principles of electrospinning processes for generating complex fibrous scaffold geometries that are similar in structural complexity to the ECM of living tissues. Qasim et al. [25] reviewed the research progress on the electrospinning of chitosan and its composite formulations for creating fibers in combination with other natural polymers to be employed in tissue engineering. The review shows that evidence exists in support of the favorable properties and biocompatibility of chitosan electrospun composite biomaterials for a range of applications. It is concluded, however, that further research and in vivo studies are required to translate these materials from the laboratory to clinical applications.
